# Sex differences in the associations of water, coffee and tea consumption with cardiovascular diseases: a prospective cohort study

**DOI:** 10.3389/fnut.2025.1530908

**Published:** 2025-01-30

**Authors:** Dandan Ke, Yueqing Wang, Yabing Hou, Weihao Shao, Jiawen Ke, Xiaoxuan Zhang, Hongxi Yang, Zhong He, Zuolin Lu

**Affiliations:** ^1^School of Population Medicine and Public Health, Chinese Academy of Medical Sciences and Peking Union Medical College, Beijing, China; ^2^School of Humanities and Social Sciences, Chinese Academy of Medical Sciences and Peking Union Medical College, Beijing, China; ^3^Department of Medical Information Technology and Management, Yanjing Medical College, Capital Medical University, Beijing, China; ^4^Department of Bioinformatics, School of Basic Medical Sciences, Tianjin Medical University, Tianjin, China

**Keywords:** water consumption, coffee consumption, tea consumption, cardiovascular disease, population-based cohort study

## Abstract

**Background:**

Water, coffee and tea are the primary sources of daily hydration. However, the sex-specific relationship between these beverages and cardiovascular disease (CVD) among remains unclear.

**Methods:**

In total, 210,239 men and 251,383 women from the UK Biobank were included. The consumption of water, coffee and tea were self-reported. CVDs, including coronary heart disease (CHD), stroke and heart failure (HF) were followed till March 1st, 2023. Sex-specific Cox models were utilized to evaluate the hazard ratios (HRs) and 95% confidence intervals (CIs) for the associations.

**Results:**

During a median follow-up of 8.7 years, 11,098 (2.40%) participants developed new-onset HF, 33,426 (7.24%) participants developed new-onset CHD, and 9,706 (2.10%) participants developed new-onset stroke. After adjustments, higher water consumption was generally associated with reduced risk of CVDs among both men and women. In contrast, heavy coffee consumption (particularly ≥6 cups/day) was associated with a greater risk of HF [1.16 (1.03–1.31) in men vs. 1.25 (1.12–1.40) in women], a greater risk of CHD [1.27 (1.18–1.36) in men vs. 1.21 (1.14–1.29) in women] and a greater risk of stroke [1.13 (0.99–1.29) in men vs. 1.20 (1.03–1.31) in women]. Similarly, heavy tea consumption was associated with an increased risk of HF (men: HR 1.19 [1.08–1.31]; women: HR 1.12 [1.02–1.23]) and CHD (men: HR 1.12 [1.05–1.18]; women: HR 1.18 [1.12–1.24]).

**Conclusion:**

Our study revealed that water consumption was associated with a lower risk of CVDs, with a lower risk of CVDs, while heavy coffee or tea consumption was linked to a higher risk. Notably, coffee and tea consumption partially attenuated the protective association of water intake with CVDs. Furthermore, significant sex differences were observed in the associations between coffee or tea consumption and CHD incidence.

## Background

Cardiovascular diseases (CVDs) are the leading cause of mortality posing substantial challenges to public health systems worldwide (1). In 2020, an estimated 19.05 million deaths were attributed to CVDs, reflecting a concerning 18.71% increase from the mortality figures recorded in 2010 (2). According to the NHANES 2017–2020 data, the prevalence of CVDs, which encompasses heart failure (HF), stroke, coronary heart disease (CHD), and hypertension, among adults was 48.6% (3). This prevalence escalates with increasing age in both sexes, thereby contributing to a significant risk of premature mortality and escalating healthcare costs.

Sexual dimorphism significantly influences the epidemiology, development, and management of CVDs in both men and women (4). Compared to men, women possess an additional X chromosome, which may result in variations in gene expression and functional outcomes within the cardiovascular system (5). Literature data suggest that women experience a two-fold incidence of CVD-related mortality compared to men (6), indicating that biological sex is a critical determinant in disease severity and resultant heterogeneity (7).

Sex hormones are known to influence behavior and lifestyle. Lifestyle behaviors, including dietary habits, have been recognized as a preventive factor in mitigating the risk of CVDs. Water, coffee and tea, being the most consumed beverages globally (8), could potentially exert significant biological effects that impact population heath. Previous studies have established a correlation between the consumption of water, coffee and tea and cardiovascular health. However, it remains uncertain whether sex could modify the aforementioned association. Therefore, this study aims to examine the potential sex differences in the association between the intake of water, coffee and tea, and the development of CVDs, utilizing data from the UK Biobank.

## Method

### Study population

The UK Biobank is a large-scale prospective cohort study that enrolled approximately 500,000 participants, aged 40 years and older, from 22 assessment centers across England, Scotland, and Wales from 2006 and 2010. All participants provided informed consent prior to their participation in this study. The study was approved by the North West Multicenter Research Ethics Committee (MREC) and the Human Tissue Authority (HTA). The study procedures were conducted in compliance with the ethical principles delineated in the World Medical Association’s Helsinki Declaration for Medical Research (9).

**Figure 1:**
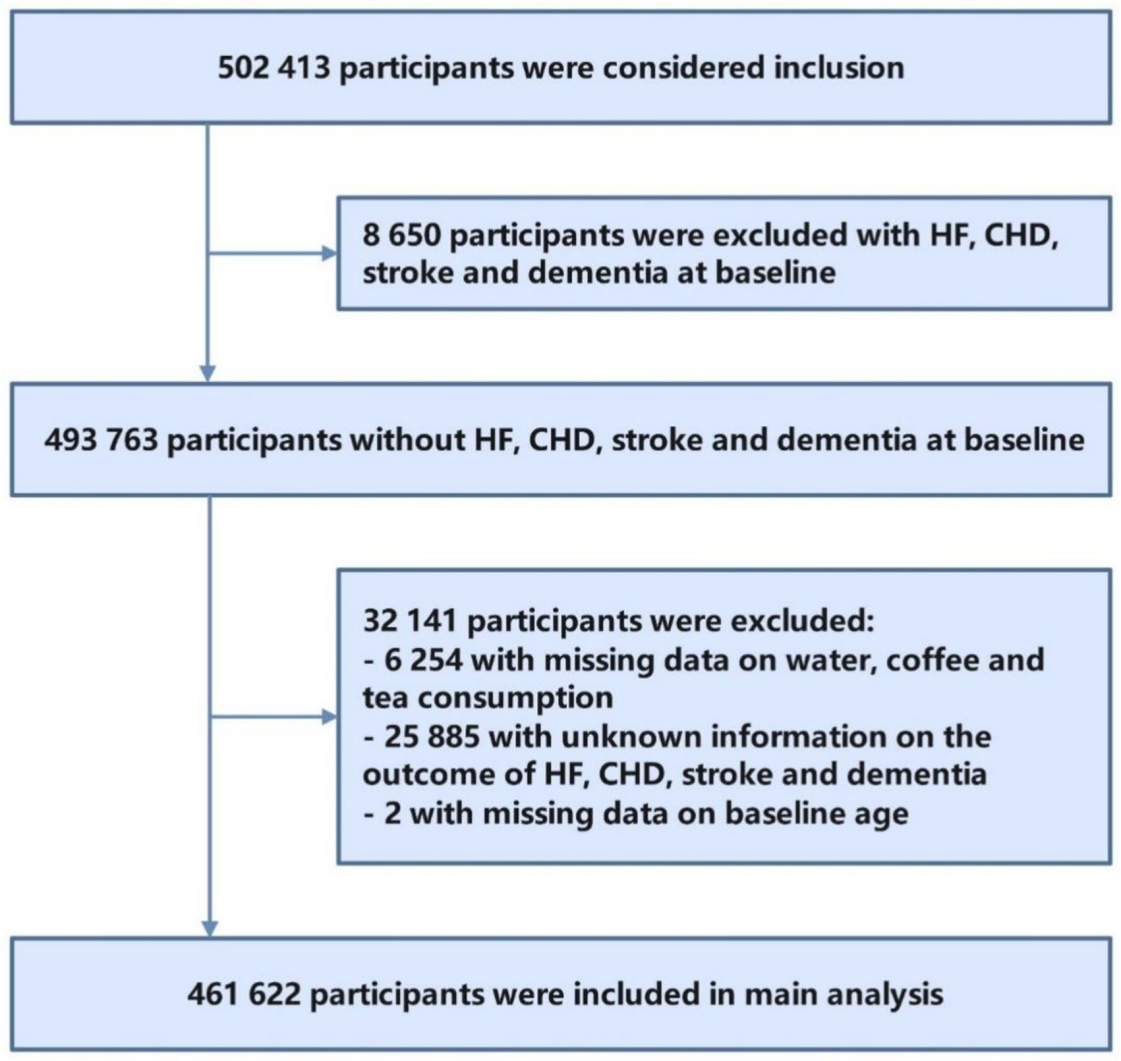
Flowchart of the study population.

The exclusion criteria were as follows: (1) had pre-existing conditions of heart failure, coronary heart disease, stroke, or dementia at baseline (*n* = 8,650); (2) had incomplete data on water, coffee or tea consumption (*n* = 6,254); (3) had missing or unknown information on the outcome of HF, CHD, stroke or dementia (*n* = 25,885); and (4) had missing information on baseline age (*n* = 2). Ultimately, 461,622 participants were included in our analyses (Figure 1).

### Exposure assessment

Based on previous studies, we chose the ACE touchscreen questionnaire to complete the assessment of exposure factors. Participants were requested to provide a daily account of their intake of water, coffee, and tea via a touchscreen questionnaire with the assistance of research personnel. The ACE touchscreen questionnaire included the following prompt for coffee intake: “Please indicate the number of cups of coffee you consume on a daily basis, including decaffeinated varieties.” Similarly, participants were asked about tea consumption: “How many cups of tea do you consume on a daily basis?” and water consumption: “How many cups of water do you drink on a daily basis?”. The Participants were instructed to provide an estimate of their average daily consumption of these beverages over the previous year. In cases of uncertainty, participants were encouraged to provide an estimate or select the “Do not know” option. Any anomalous responses, such as “<0 cups/day” and “>99 cups/day,” were excluded from the analysis. If a participant reported an intake of “>10 cups/day,” they were prompted to verify this information. Additionally, a composite variable was created to aggregate the daily intake of coffee and tea for each participant.

**Figure 2:**
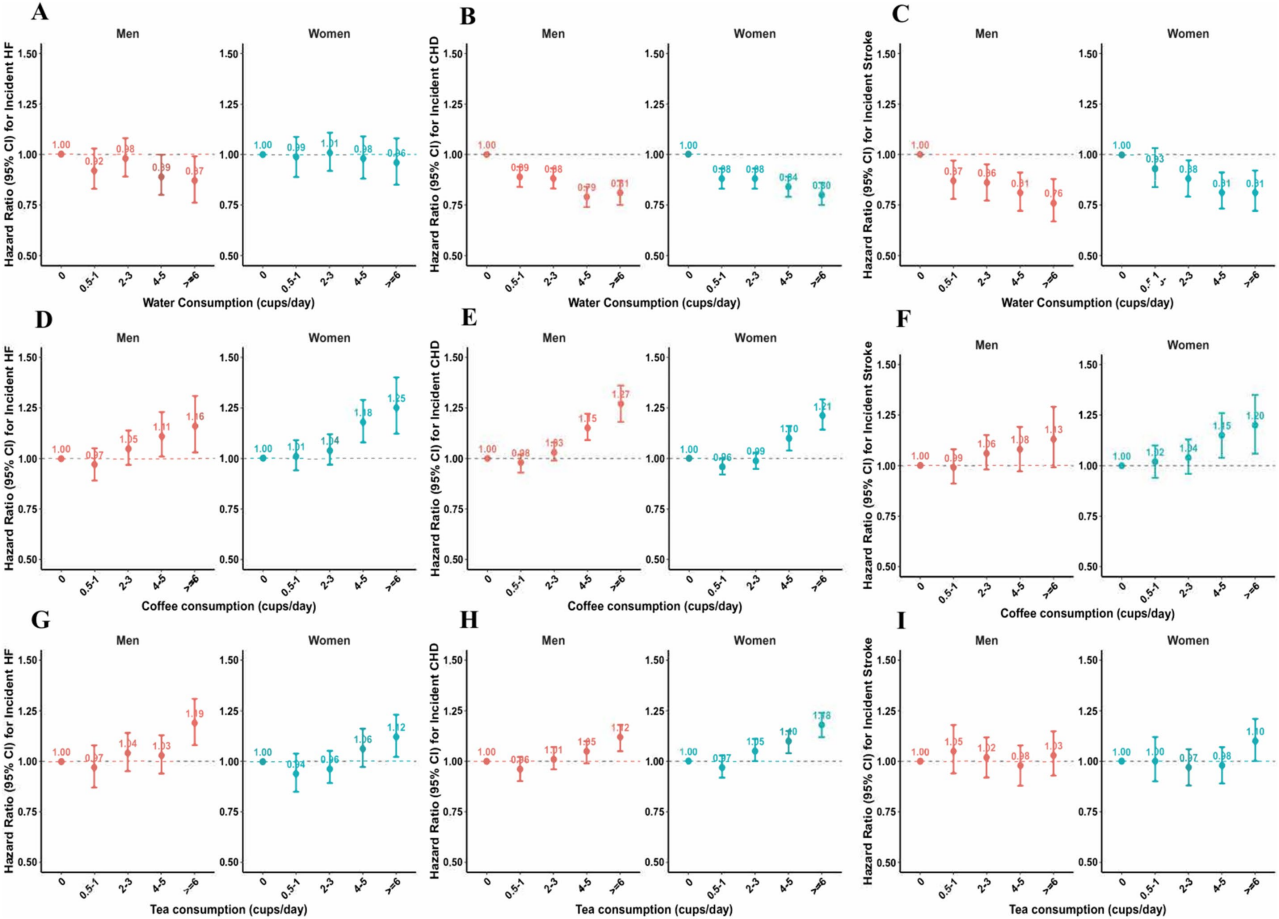
Association of water, coffee and tea intake with cardiovascular diseases among men and women. **(A–C)** Associations of water with HF, CHD and Stroke events in fully-adjusted model. **(D–F)** Associations of coffee with HF, CHD and Stroke events in fully-adjusted model. **(G–I)** Associations of tea with HF, CHD and Stroke events in fully-adjusted model. The multivariable model was adjusted for baseline age, ethnicity, education, income, smoking status, physical activity, diet pattern, body mass index, systolic blood pressure, diastolic blood pressure, triglycerides, high-density lipoprotein, low-density lipoprotein, long-standing illness, disability or infirmity, Townsend Deprivation Index, alcohol consumption and milk consumption.

The reported consumption varied from “0 cups/day” to “99 cups/day.” Responses such as “Do not know” and “Prefer not to answer” were excluded from the analysis. Adhering to established protocols from previous studies, We categorized the daily consumption of water, coffee and tea into five distinct groups (none, 0.5-1 cup/day, 2–3 cups/day, 4–5 cups/day, and ≥6 cups/day). Similarly, the daily consumption of the composite variable was categorized into five groups (none, 0.5-2 cup/day, >2–4 cups/day, >4–8 cups/day, and >8 cups/day) (Figure 2).

### Outcome assessment

New-onset CVDs, encompassing incident cases of HF, stroke and CHD, were identified from hospital admission, primary care and/or death registry data linked to the UK Biobank (9). Diagnoses were determined using the International Classification of Diseases-10th Revision (ICD-10) coding system. Specifically, HF was defined as ICD-10 codes: 150; CHD was defined as ICD-10 codes: I20-22, 24, 25; and stroke was defined as ICD-10 codes: I60-64. Follow-up ended on March 31, 2021. The participants were censored at the end of the follow-up period, the date of death, or loss to follow-up, whichever occurred first.

### Assessment of covariates

Age, ethnic (White, Black or Black British, and other ethnic groups), education (university or college educational level, other), average pre-tax income (less than £18,000, £18,000 to £30,999, £31,000 to £51,999, £52,000 to £100,000, greater than £100,000), smoking status (never, previous, and current), physical activity, alcohol consumption, milk consumption (daily milk intake, including full cream, semi-skimmed, skimmed, soya or other types of milk; never/rarely drink milk) and dietary pattern [healthy or unhealthy, healthy diet was based on consumption of at least 4 out of 7 dietary components: (1) fruit: ≥3 portions per day; (2) vegetables: ≥3 portions per day; (3) fish: ≥2 portions/week; (4) processed meat: ≥1 portion/week; (5) unprocessed red meat: ≥1.5 servings/week; (6) whole grains: ≥3 servings/day; (7) refined grains: ≥1.5 servings/day (10, 11)] were self-reported during the interview process. Height, weight, and blood pressure were measured at the assessment center, with BMI calculated as weight in kilograms divided by height in meters squared. Blood pressure was measured twice using an Omron 705 IT electronic monitor, and average levels were utilized. Serum cholesterol was measured in the central laboratory. Townsend Deprivation Index (TDI), reflecting socioeconomic status with scores inversely related to socioeconomic status, was defined according to participant’s postcode, using a combination of unemployment, noncar ownership, nonhome ownership, and household overcrowding (12).

### Statistical analysis

Baseline characteristics were stratified by sex. Continuous variables are presented as the mean and standard deviation (SD), while categorical variables are expressed as percentages (%). To assess the mediating role of sex differences to the association between water, coffee and tea consumption and CVDs, exposure factors were converted into numerical factors for analysis.

The reference group in our study consisted of individuals who did not consume water, coffee, or tea daily. We employed Cox proportional hazard regression models to assess the associations between water, coffee and tea intake and the incidence of CVDs, including HF, stroke and CHD. The findings from these models were presented as hazard ratios (HRs) with corresponding 95% confidence intervals (CIs). The multivariable models underwent multiple rounds of adjustment. In the first model, adjustments were made for baseline age and ethnicity. In the fully adjusted model, additional potential confounders were incorporated. These included factors such as educational qualification, employment status, income, smoking habits, physical activity patterns, dietary patterns, body mass index (BMI), systolic and diastolic blood pressure, triglyceride levels, LDL-cholesterol, HDL-cholesterol, presence of long-term illness, and consumption patterns of alcohol, water, tea, coffee, and milk.

To enhance the robustness of our study results, we conducted several sensitivity analyses. First, we excluded individuals with long-standing illnesses, disabilities, or infirmities at baseline and re-analyzed the remaining participants. Second, we performed a stratified analysis by age (categorized at 60 years) to explore potential variations in the effects based on baseline age. Third, we repeated our main analysis in the 375,094 containing coffee consumed type (caffeinated coffee or decaffeinated coffee). In our analysis, we performed multiple imputation through chained equations with 5 iterations to manage missing values, which were less than 20%. Detailed information regarding missing data is provided in Supplementary Table S1. Two-sided *p*-value < 0.05 was considered statistically significant. All analyses were performed using the R software package, version 4.3.2.

## Results

### Baseline characteristics

A total of 461,622 participants were included in our study. Among the 461,622 participants, 251,383 (54.42%) were women, and 210,239 (45.58%) were men. The average age at baseline for women was 56.34 years (SD ± 8.00) and 56.73 years (SD ± 8.20) for men. Compared to women, men were more likely to be older, of White ethnicity, have higher educational attainment, maintain more stable employment, demonstrate lower TDI (indicating a higher socioeconomic status), exhibit higher BMI and triglyceride levels, engage in current smoking, and report a higher frequency of alcohol consumption. Further details are provided in Table 1. During a median follow-up period of 8.71 years for new-onset CVDs (especially HF, CHD and stroke), 11,098 participants (2.40%) developed HF, 33,426 patients (7.24%) developed the CHD and 9,706 patients (2.10%) developed stroke.

**Table 1:** Baseline characteristics of the study population.

Characteristic	Women	Men	*p* values
Number (*n*, %)	251,383 (54.4)	210,239 (45.6)	–
Age (years)	56.34 (8.00)	56.73 (8.20)	<0.001
Ethnic (*n*, %)			<0.001
White	226,823 (90.2)	190,487 (90.6)	
Black or Black British	23,419 (9.3)	18,396 (8.8)	
Other	1,141 (0.5)	1,356 (0.6)	
Body mass index (kg/m^2^)	27.0 (5.1)	27.8 (4.2)	<0.001
Systolic blood pressure (mmHg)	137.2 (20.2)	142.7 (18.5)	<0.001
Diastolic blood pressure (mmHg)	80.7 (10.5)	84.0 (10.5)	<0.001
LDL-cholesterol (mmol/L)	3.62 (0.87)	3.48 (0.86)	<0.001
HDL-cholesterol (mmol/L)	1.59 (0.37)	1.28 (0.32)	<0.001
Townsend deprivation index	−1.38 (3.04)	−1.35 (3.06)	<0.01
Education, university or college (*n*, %)	77,713 (30.9)	70,499 (33.5)	<0.001
Employment (*n*, %)			<0.001
Working	139,731 (55.6)	127,738 (60.8)	
Retired	87,732 (34.9)	65,435 (31.1)	
Unemployment	21,093 (8.4)	14,655 (7.0)	
None of the above	2,827 (1.1)	2,411 (1.1)	
Income (*n*, %)			0.67
Less than £18,000	54,807 (21.8)	45,719 (21.7)	
£18,000 to £30,999	63,668 (25.3)	53,318 (25.4)	
£31,000 to £51,999	66,753 (26.6)	55,532 (26.4)	
£52,000 to £100,000	52,141 (20.7)	43,831 (20.8)	
£100,000 and above	14,014 (5.6)	11,839 (5.6)	
Smoking status (*n*, %)			<0.001
Never	149,897 (59.6)	103,051 (49.0)	
Previous	78,899 (31.4)	80,773 (38.4)	
Current	22,587 (9.0)	26,415 (12.6)	
Healthy physical activity (*n*, %)	159,312 (63.4)	133,496 (63.5)	0.39
Healthy diet pattern (*n*, %)	51,217 (20.4)	42,355 (20.1)	0.06
Alcohol consumption (*n*, %)			<0.01
Never	19,467 (7.7)	15,891 (7.6)	
Special occasions only	56,950 (22.7)	47,083 (22.4)	
One to four times a week	123,736 (49.2)	104,058 (49.5)	
Daily or almost daily	51,230 (20.4)	43,207 (20.6)	
Water consumption (cups/d)	2.74 (2.25)	2.73 (2.27)	0.095
Coffee consumption (cups/d)	2.01 (2.06)	2.02 (2.08)	0.008
Tea consumption (cups/d)	3.40 (2.85)	3.41 (2.86)	0.260
Have milk consumption (*n*, %)	243,041 (96.70)	203,236 (96.70)	0.819

Values are shown as mean (standard deviation) for continuous variables and number (percentage) for categorical variables. Difference between women and men are compared using Student’s *t* test or chi-square test accordingly. LDL-cholesterol, low-density lipoprotein cholesterol; HDL-cholesterol, high-density lipoprotein cholesterol.

### Water consumption and cardiovascular disease risk

Our findings suggest that water consumption is associated with a decreased risk of CVDs incidence (Figure 2). After adjusting for ethnicity and baseline age, we found that individuals who consumed ≥6 cups/day of water were associated with a reduced risk of HF in both sexes [HR (95% CI): 0.73 (0.64–0.82) in men; 0.82 (0.73–0.92) in women]. However, after comprehensive adjustment, we found a negative association among men, not women. Compared to non-water drinkers, the HRs (95% CIs) for consuming ≥6 cups of water per day were 0.87 (95% CIs: 0.76–0.99) in men and 0.96 (95% CIs: 0.85–1.08) in women. The *p* value for the sex interaction was 0.14 in Model 1 and 0.22 in fully adjusted Model, highlighting a moderate sex difference.

Likewise, water consumption was associated with a lower incidence of CHD in both men and women, as observed in Model 1 and Model 2. As shown in Supplementary Table S2, the results from the multivariate Cox model (Model 2) showed that water consumption was associated with a reduced incidence of CHD. Those who consumed who drank ≥6 cups of water per day were associated with a 20% lower incidence of CHD compared with those who did not consume water [HR (95% CI): 0.81 (0.75–0.87) in men; HR (95% CI): 0.80 (0.75–0.86) in women], all p for trend < 0.001. A similar association was observed between water consumption and stroke incidence in both men and women. After adjusting for confounders in Model 2, compared to non-water drinkers, people who consumed ≥6 cups/day of water were associated with a lower risk of stroke [HRs (95CIs): 0.76 (0.67–0.88) in men; 0.81 (0.72–0.92) in women], all p for trend <0.001.

### Coffee consumption and cardiovascular disease risk

Heavy coffee consumption was associated with a higher risk of CVDs incidents in both men and women (Figure 2). As shown in Supplementary Table S3, after full adjustment, we observed that men and women who consumed ≥6 cups/day of coffee were associated with a higher risk of HF [HR (95% CI): 1.16 (1.03–1.31) in men, p for trend = 0.001; 1.25 (1.12–1.40) in women, p for trend = 0.001]. After adjusting for ethnicity and baseline age, we found that moderate coffee consumption was associated with a lower risk of CHD, particularly among those who consumed 0.5–3 cups/day of coffee. Nevertheless, in the fully-adjusted model, we find the positive associations both in men and women. Compared with non-coffee drinkers, men who consumed ≥6 cups/day of coffee per day were associated with a 27% increase in CHD events [HR (95% CI): 1.27 (1.18–1.36), p for trend <0.001] and a 13% increase in stroke events [HR (95% CI): 1.13 (0.99–1.29), p for trend = 0.02]. In the women’s group, compared with non-coffee consumers, We observed a 21 and 20% increase in the incidence of CHD and stroke, respectively, associated with those who consumed ≥6 cups of coffee per day. Additionally, we found a statistically significant gender difference between coffee consumption and incident CHD, with a *p*-value < 0.05.

### Tea consumption and cardiovascular disease risk

Heavy tea consumption was associated with a higher risk of HF and CHD in both men and women (Figure 2). As shown in Supplementary Table S4, after adjusting for ethnicity and baseline age, compared to non-tea drinkers, men who consumed 0.5–1 cups of tea per day were associated with a 12% reduced risk of incident HF [HR (95% CI): 0.88 (0.79–0.98)], and women who consumed 0.5–1 cups of tea per day were associated with a 15% reduced risk of incident HF [HR (95% CI): 0.85 (0.77–0.94)]. In Model 2, we found a positive association between tea consumption and HF incidence among men and women. After full adjustment, we found that those who drank ≥6 cups of tea per day were associated with a higher risk of HF compared to the reference [HR (95% CI): 1.19 (1.08–1.31) in men; HR (95% CI): 1.12 (1.02–1.23) in women, all p for trend <0.001].

In our study, we found that moderate tea consumption was associated with a lower risk of CHD incidence among men and women (Model 1). After adjusting for ethnicity and baseline age, compared to those who did not drink tea daily, the HRs (95% CIs) for drinking tea 0.5–1 cup/day and 2–3 cups/day were 0.90 (0.84–0.95) and 0.93 (0.89–0.98) among men, respectively; the HRs (95% CIs) for drinking tea 0.5–1 cup/day and 2–3 cups/day were 0.91 (0.86–0.97) and 0.98 (0.94–1.03) among women, respectively. According to Model 2, heavy tea consumption was associated with a higher risk of CHD in both men and women. Compared to non-tea drinkers, women who consumed ≥6 cups/day of coffee were associated with a 18% increased risk of CHD [HR (95% CI): 1.18 (1.12–1.24), p for trend < 0.001]; men who consumed ≥6 cups/day of coffee had a 12% increased risk of CHD [HR (95% CI): 1.12 (1.05–1.18), p for trend < 0.001]. Additionally, we found a statistically significant gender difference between tea consumption and incident CHD, with a *p*-value < 0.05.

### Coffee and tea consumption and cardiovascular disease risk

Our results revealed that heavy coffee and tea consumption was associated with a higher risk of HF and CHD. As shown in Supplementary Table S5, the results from the multivariate Cox model (Model 3) showed that compared to non-coffee or non-tea drinkers, people who consumed more than eight cups per day were associated with a greater risk for incident HF [HRs (95% CI): 1.48 (1.20–1.84) in men; 1.48 (1.22–1.80) in women, with all p for trend < 0.001].

Similarly, results from the multivariate Cox model (Model 3) showed that compared to those who did not drink coffee or tea per day, those who consumed more than 8 cups of coffee and tea on a daily basis were associated with approximately 48% higher probability of experiencing a CHD event [HRs (95% CI): 1.48 (1.30–1.69) in men; 1.49 (1.33–1.68) in women, with all p for trend < 0.001].

### Stratified analysis

**Figure 3:**
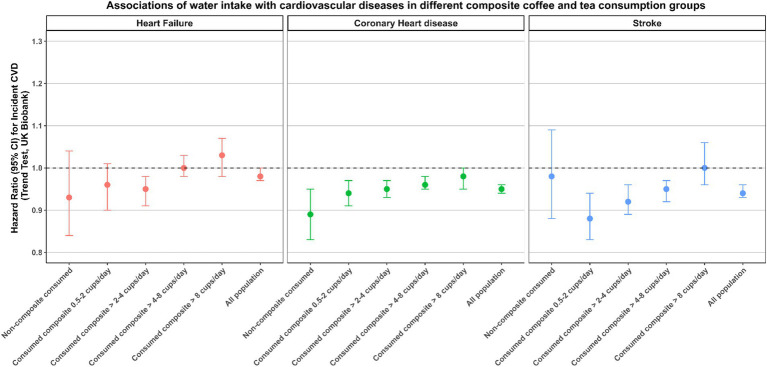
Associations of water intake with CVD incidence in different composite coffee and tea consumption groups. The multivariable model was adjusted for baseline age, ethnicity, education, income, smoking status, physical activity, diet pattern, body mass index, systolic blood pressure, diastolic blood pressure, triglycerides, high-density lipoprotein, low-density lipoprotein, long-standing illness, disability or infirmity, Townsend Deprivation Index, alcohol consumption, milk consumption, coffee consumption and tea consumption.

**Figure 4:**
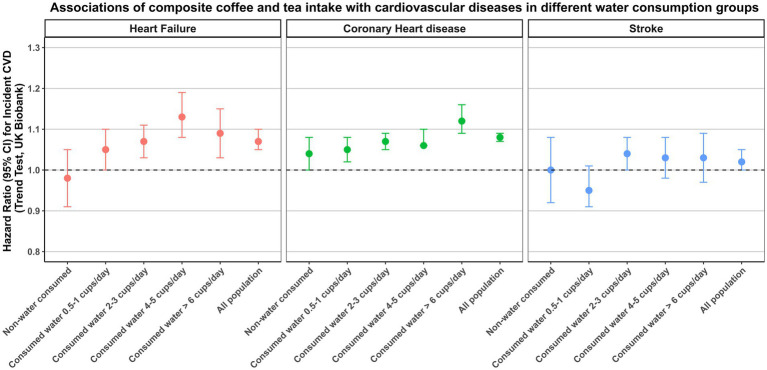
Associations of composite coffee and tea intake with cardiovascular diseases in different water consumption groups. The multivariable model was adjusted for baseline age, ethnicity, education, income, smoking status, physical activity, diet pattern, body mass index, systolic blood pressure, diastolic blood pressure, triglycerides, high-density lipoprotein, low-density lipoprotein, long-standing illness, disability or infirmity, Townsend Deprivation Index, alcohol consumption and milk consumption.

The results of our study indicated a negative association between daily water consumption and the incidence of CVD, while a positive association was observed between daily coffee and tea consumption and the incidence of CVD. Consequently, we devised a composite beverage consumption, representing the sum of participants’ daily coffee and tea consumption. The Spearman correlation coefficients between daily water consumption and composite consumption was calculated, yielding a value of −0.24. We used this composite consumption to ascertain whether the inverse correlation between daily water consumption and cardiovascular incidents diminished in different populations due to increase consumption of the composite variable. Additionally, we evaluated the impact of the positive correlation between daily coffee and tea consumption and cardiovascular incidents in the context of increased daily water consumption. As illustrated in Figures 3, 4, the results obtained for these two sets of associations do not diverge in opposite directions. The inverse association between water consumption and the incidence of CVD, as illustrated in Figure 3, exhibited a decreasing trend as the daily intake of coffee and tea increased.

### Sensitivity analysis

The findings of the primary analyses were consistent in individuals aged 60 years and older, in a control group of individuals without long-term illness, and in the caffeine-only population. The daily consumption of water, coffee, and tea did not significantly differ between the sexes among individuals aged 60 years and above (Supplementary Table S6). Compared to non-water drinkers, individuals aged 60 years and above had a decreased risk of cardiovascular events associated with increased daily water intake (Supplementary Table S7). Compared to non-coffee drinkers, there was an increased risk of stroke and CHD incidence associated with increased daily coffee intake among individuals aged 60 years and above (Supplementary Table S8). Compared to non-tea drinkers, there was an increased risk of HF and CHD incidence associated with increased daily tea intake among individuals aged 60 years and above (Supplementary Table S9). We found a statistically significant difference between sexes in coffee and tea consumption and in the incidence of CHD events among participants aged 60 years and above (Supplementary Tables S8, S9). Similarly, we replicated the findings of the main analysis in the population without long-term illness population and found no violations of the hypothesis (Supplementary Tables S12–S14). Additionally, we repeated our main analysis in the caffeine-only population, and we found that statistically significant associations between water and coffee consumption and incident CVD remained, consistent with the results of the main analysis. The statistical significance between tea consumption and CVD event incidence became weaker in this population (Supplementary Tables S16–S20).

## Discussion

In this large prospective cohort study, we investigated the relationship between the consumption of water, coffee and tea, and the incidence of CVDs among both sexes. The main findings of the study are as follows: (1) enough daily water intake is associated with reduced risk of the incidence of HF, CHD and stroke; (2) high coffee consumption is associated with a greater risk of HF, CHD and stroke among both sexes; (3) heavy tea consumption is associated with a greater risk of HF and CHD in both sexes; and (4) stable positive association emerged between daily coffee and tea consumption and cardiovascular events, regardless of water consumption; and (5) statistically significant gender differences have been found with regard to coffee and tea consumption and the subsequent incidence of CHD.

### Water consumption with CVDs

Several studies have investigated the link between water consumption and cardiovascular diseases, but the findings have been inconsistent. In the Adventist Health Study, a prospective examination involving 8,280 men and 12,017 women, higher water consumption was negatively associated with the risk of fatal CHD, with the negative association with water stronger in men (13). Additionally, during a large prospective study initiated in 1988–1990 involving 46,465 men and 64,327 women, they found that water intake from foods and beverages was associated with reduced risk of mortality from CHD and total CVD with higher reduced risk of mortality in women (14). These findings underscore the potential role of water consumption as a protective factor against cardiovascular diseases (13–18). However, some studies revealed a positive or no association between water consumption and cardiovascular disease (19–24). Two cross-sectional studies, one using NHANES 2005–2006 data (19) and the other using KNHANES 2012 data (21), found no statistically significant associations between water intake and cardiovascular diseases. However, it is important to note that both studies relied on observational data and utilized cross-sectional analysis. As a result, they may only provide minimal information for making causal inferences.

In the present study, we found a significant association between water consumption and a reduced risk of both CHD and stroke among men and women. Notably, our research benefits from its reliance on prospective cross-sectional data sourced from a large population, which enhances the robustness of our conclusions. The findings of our present study may be explained by several potential biological mechanisms. Inadequate water intake is related to a reduced risk of inflammation (18) and increased blood viscosity (25), which are major determinants of atherosclerosis and stroke (26, 27). Additionally, chronic dehydration may lead to elevated levels of hemorrhagic factors, which, consequently, are associated with increased levels of coagulation factors, blood viscosity, fibrinogen, arterial stiffness and hematocrit (28, 29). These factors are correlated with arterial thrombosis, thus contributing to the development of both CHD and stroke (30, 31).

However, it is equally important to consider the risks associated with excessive water consumption. Overhydration may lead to hyponatraemia (low sodium levels), a potentially life-threatening condition characterized by symptoms such as nausea, confusion, and seizures (32). Hyponatremia has been associated with adverse cardiovascular outcomes, including arrhythmias and heart failure, due to the resulting electrolyte imbalance (33). Further research is needed to establish an optimal range of water consumption for cardiovascular health, balancing the risks of dehydration and overhydration.

### Coffee consumption with CVDs

Our study revealed a positive association between high coffee intake and the risk of heart failure, CHD, and stroke among both sexes. This correlation was particularly significant for individuals who consumed four or more cups of coffee per day. Several case–control and prospective studies have revealed that the development of CVD may be associated with coffee intake (34–37). Zhou et al. reported that among 347,077 individuals from the UK Biobank, those who consumed >6 cups/day of coffee had a multivariable RR of 1.22 (95% CI: 1.07–1.40) for the incidence of CVD compared with those who consumed 1–2 cups/day (38). Chen et al. reported that compared with noncoffee drinkers, people who drank ≥6 cups of coffee per day had greater risks of CVD, CHD and stroke, with HRs and 95% CIs of 1.03 (0.98–1.09), 1.04 (0.98, 1.10) and 1.02 (0.92, 1.14), respectively (39). Our findings are consistent with the results of these prospective studies, all of which used data from the UK Biobank database.

The increased risk of cardiovascular disease associated with heavy coffee consumption can be explained by the following potential biological mechanisms. First, the most commonly consumed type of coffee in the UK is instant coffee, which contains dairy products and sugar. The high consumption of coffee may increase the burden on the cardiovascular circulatory system, potentially leading to cardiovascular events. Additionally, coffee contains a diverse array of bioactive compounds, including caffeine, CGA, diterpene alcohols, minerals such as potassium and magnesium, niacin and lignans (40). High short-term coffee intake may dramatically increase plasma renin activity, catecholamine concentrations and blood pressure, increase vascular tension, and induce cardiac arrhythmias (41).

### Tea consumption with CVDs

In our study, we found that individuals who consumed ≥6 cups/day of tea were associated with a moderate increase in the risk of HF and coronary heart disease both in men and women. Few previous studies reported findings consistent with our current analysis. Previous studies have reported that tea, a popular lifestyle component, can promote cardiovascular health due to its antioxidant effects and anti-inflammatory mechanisms (42, 43). The results from a systematic review showed that moderate tea consumption was associated with a lower risk of coronary heart disease, but a large amount (>4–6 cups/day) of tea consumption has been shown to increase the risk of coronary heart disease (44).

In our study, we also found that the consumption of 0.5–1 cup/day tea was associated with a lower risk of CHD in both men and women, but fully adjusted models were not used. Interestingly, research has shown that tea consumption may be associated with a lower risk of coronary heart disease in Japanese (45), Saudi Arabian (46), and Chinese adults (47, 48), but similar results have rarely been found in the British population. The type of tea consumed and the tea-drinking habits of the British people may explain this difference. For example, the British population prefers to drink black tea with milk and sugar, but previous studies have reported that black and green tea may have inconsistent biological effects on coronary heart disease risk. Green tea catechins have been shown to inhibit oxidation, vascular inflammation, atherogenesis and thrombogenesis and favorably modulate the plasma lipid profile and vascular reactivity, suggesting a broad spectrum of beneficial effects on CHD (49). Similarly, excessive consumption of milk and sugar also increases the risk of coronary heart disease.

### Sex interaction between fluid consumption and cardiovascular diseases

The focus on the sex-specific epidemiology, manifestation, pathophysiology, treatment and outcome of major chronic diseases has been increasing steadily, and the interaction of sex differences in disease development needs to be considered. In our study, the sex interaction effect was statistically significant only for the associations between coffee and tea consumption and the incidence of CHD. However, we did not find inconsistent results regarding fluid consumption or coronary heart disease among men and women. We also found that the hazard ratios for the same dose of fluid intake were greater in women than in men for the same exposure factors and study endpoints. However, it is important to note that these findings are statistically significant, and further research is necessary to validate this conclusion.

### Strengths and limitations

The strengths of our study include its prospective design, large sample population, long follow-up time, and well-validated covariate information. However, several potential limitations still should be considered. First, information on water, coffee and tea consumption was derived from self-reported questionnaires at baseline, which may have recall bias and may not reflect consumption patterns over time, leading to potential misclassifications of the exposures. Second, although we adjusted for confounders such as coffee and tea intake in investigating the association between water consumption and major chronic diseases, we did not account for other confounders (e.g., sugar intake, beverage additives, other sources of water intake) due to experimental design limitations, which may have affected the accuracy of our findings. Third, due to the lack of data on tea type (black tea, green tea), we were unable to investigate whether the type of tea consumed affects the association between tea consumption and cardiovascular disease incidence. Future studies are needed to investigate the association of tea consumed type in incident CVDs in the general population. Forth, participants in the UK Biobank cohort tended to be more health conscious than nonparticipants, and a large number of them were White people. Consequently, this may diminish the generalisability of the findings to more heterogeneous demographics or regions. It is therefore essential to exercise caution when generalizing these findings to other populations.

## Conclusion

In our study, we found that high coffee and tea consumption was associated with an increased risk of cardiovascular diseases in both sexes (especially when they consume ≥6 cups/day); daily water consumption was associated with a lower risk of cardiovascular diseases in both men and women. Our research indicates the significance of moderate fluid consumption in daily life.

## Data Availability

The original contributions presented in the study are included in the article/Supplementary material, further inquiries can be directed to the corresponding authors.
