# Supplementary material for: Sex differences in the associations of water, coffee and tea consumption with cardiovascular diseases: a prospective cohort study
Source: Front Nutr. 2025 Jan 30;12:1530908. doi: 10.3389/fnut.2025.1530908 (PMC11823423; doi:10.3389/fnut.2025.1530908)
Supplement: Supplementary file 1 [file Table_1.docx]

**Supplementary materials online only for the manuscript:**

**Title: Sex differences in the associations of water, coffee and tea consumption with cardiovascular diseases: a prospective cohort study**

**Authors:** Dandan Ke^1,2*^, Yueqing Wang, MSc^1*^, Yabing Hou, PhD^3^, Weihao Shao, MSc^1^, Jiawen Ke^1^, Xiaoxuan Zhang, MSc^1^, Hongxi Yang, PhD^4^, Zhong He, PhD^2†^, Zuolin Lu, PhD^1†^

^1^ School of Population Medicine and Public Health, Chinese Academy of Medical Sciences & Peking Union Medical College, Beijing, China.

^2^ School of Humanities and Social Sciences, Chinese Academy of Medical Sciences & Peking Union Medical College, Beijing, China.

^3^ Department of Medical Information Technology and Management, Yanjing Medical College, Capital Medical University, Beijing, China.

^4^ Department of Bioinformatics, School of Basic Medical Sciences, Tianjin Medical University, Tianjin, China.

*Contributed equally.

**Correspondence to:**

Zuolin Lu, MSc, PhD

Address: School of Population Medicine and Public Health, Chinese Academy of Medical Sciences & Peking Union Medical College, 100730 Beijing, China.

Email: luzuolin@cams.cn

Tel: +86 195 2038 0053

Zhong He, PhD

Address: School of Humanities and Social Sciences, Chinese Academy of Medical Sciences & Peking Union Medical College, 100730 Beijing, China.

Email: hezhong@shss.pumc.edu.cn

**Table S1.** Detailed information on missing covariates

| Covariates | N* | Missing rate (%) |
| --- | --- | --- |
| Alcohol consumption | 352 | 0.07 |
| Income | 72390 | 14.85 |
| Smoking status | 2821 | 0.58 |
| Physical activity | 68850 | 14.12 |
| Diet pattern | 24965 | 5.12 |
| BMI | 2997 | 0.61 |
| SBP | 33406 | 6.85 |
| DBP | 33393 | 6.85 |
| Triglycerides | 32253 | 6.62 |
| LDL | 32749 | 6.72 |
| HDL | 70405 | 14.44 |
| Longtime illness | 11539 | 2.37 |
| TDI | 604 | 0.12 |

Abbreviations: BMI, body mass index (calculated as weight in kilograms divided by height in meters squared); HDL, high density lipoprotein; LDL, low density lipoprotein; SBP, systolic blood pressure; DBP, diastolic blood pressure; TDI, Townsend Deprivation Index (a factor according to participant’s postcode, using a combination of unemployment, non-car ownership, non-home ownership, and household overcrowding reflects socioeconomic status whose scores are reversely with socioeconomic status).

*N represents the number of missing responses.

**Table S2**. Hazard ratio (95% CIs) for cardiovascular diseases according to water intake among men and women in the UK Biobank

| Outcomes | Non-consumers | Water intake (cups per day) | | | | p for trend | p for sex interaction |
| --- | --- | --- | --- | --- | --- | --- | --- |
|  |  | 0.5-1 | 2-3 | 4-5 | ≥6 |  |  |
|  | (*n*=37,050) | (*n*=114,223) | (*n*=175,135) | (*n*=84,765) | (*n*=50,449) |  |  |
| **Incident heart failure (n = 11,098)** | | | | | | | |
| Men |  |  |  |  |  |  |  |
| Model 1^a^ | 1.00 (*ref*) | 0.82 (0.74-0.91) | 0.85 (0.77-0.94) | 0.76 (0.68-0.84) | 0.73 (0.64-0.82) | ＜0.001* | 0.14 |
| Model 2^b^ | 1.00 (*ref*) | 0.93 (0.83-1.03) | 0.98 (0.89-1.08) | 0.89 (0.80-1.00) | 0.87 (0.77-0.99) | 0.05* |  |
| Women |  |  |  |  |  |  |  |
| Model 1^a^ | 1.00 (*ref*) | 0.88 (0.80-0.97) | 0.89 (0.81-0.98) | 0.84 (0.76-0.94) | 0.82 (0.73-0.92) | ＜0.01* | 0.23 |
| Model 2^b^ | 1.00 (*ref*) | 0.99 (0.90-1.09) | 1.01 (0.92-1.11) | 0.98 (0.88-1.09) | 0.96 (0.86-1.08) | 0.58 |  |
| **Incident coronary heart disease (n =33,426)** | | | | | | | |
| Men |  |  |  |  |  |  |  |
| Model 1^a^ | 1.00 (*ref*) | 0.82 (0.78-0.87) | 0.79 (0.75-0.84) | 0.70 (0.66-0.75) | 0.70 (0.66-0.76) | ＜0.001* | 0.14 |
| Model 2^b^ | 1.00 (*ref*) | 0.89 (0.84-0.95) | 0.88 (0.83-0.93) | 0.79 (0.74-0.85) | 0.81 (0.75-0.87) | ＜0.001* |  |
| Women |  |  |  |  |  |  |  |
| Model 1^a^ | 1.00 (*ref*) | 0.82 (0.78-0.87) | 0.81 (0.77-0.85) | 0.75 (0.71-0.80) | 0.71 (0.67-0.76) | ＜0.001* | 0.25 |
| Model 2^b^ | 1.00 (*ref*) | 0.88 (0.84-0.94) | 0.88 (0.84-0.93) | 0.84 (0.79-0.89) | 0.80 (0.75-0.86) | ＜0.001* |  |
| **Incident stroke (n = 9,706)** | | | | | | | |
| Men |  |  |  |  |  |  |  |
| Model 1^a^ | 1.00 (*ref*) | 0.81 (0.73-0.90) | 0.79 (0.71-0.87) | 0.73 (0.65-0.82) | 0.69 (0.60-0.78) | ＜0.001* | 0.79 |
| Model 2^b^ | 1.00 (*ref*) | 0.88 (0.79-0.98) | 0.86 (0.78-0.96) | 0.81 (0.72-0.91) | 0.77 (0.67-0.88) | ＜0.001* |  |
| Women |  |  |  |  |  |  |  |
| Model 1^a^ | 1.00 (*ref*) | 0.87 (0.78-0.96) | 0.82 (0.74-0.90) | 0.75 (0.67-0.83) | 0.74 (0.66-0.84) | ＜0.001* | 0.92 |
| Model 2^b^ | 1.00 (*ref*) | 0.93 (0.84-1.03) | 0.88 (0.80-0.97) | 0.82 (0.73-0.91) | 0.82 (0.72-0.93) | ＜0.001** |  |

Model 1 was adjusted for ethnicity and baseline age. Model 2 was additionally adjusted for qualification, employment, income, smoking status, physical activity pattern, diet pattern, body mass index, systolic blood pressure, diastolic blood pressure, triglycerides, LDL-cholesterol, HDL-cholesterol, long-term illness status, alcohol consumption.

| Outcomes | Non-consumers | Coffee intake (cups per day) | | | | p for trend | p for sex interaction |
| --- | --- | --- | --- | --- | --- | --- | --- |
|  |  | 0.5-1 | 2-3 | 4-5 | ≥6 |  |  |
|  | (*n*=101,542) | (*n*=126,306) | (*n*=143,965) | (*n*=61,786) | (*n*=28,023) |  |  |
| **Incident heart failure (n = 11,098)** | | | | | | | |
| Men |  |  |  |  |  |  |  |
| Model 1^a^ | 1.00 (*ref*) | 0.90 (0.83-0.97) | 0.92 (0.85-0.99) | 0.97 (0.88-1.06) | 1.09 (0.97-1.23) | 0.46 | 0.46 |
| Model 2^b^ | 1.00 (*ref*) | 0.96 (0.89-1.04) | 1.03 (0.95-1.11) | 1.05 (0.96-1.16) | 1.09 (0.97-1.23) | 0.03* |  |
| Women |  |  |  |  |  |  |  |
| Model 1^a^ | 1.00 (*ref*) | 0.94 (0.87-1.01) | 0.91 (0.85-0.98) | 1.02 (0.94-1.11) | 1.18 (1.06-1.32) | 0.06 | 0.47 |
| Model 2^b^ | 1.00 (*ref*) | 1.01 (0.94-1.09) | 1.02 (0.95-1.10) | 1.13 (1.04-1.23) | 1.18 (1.06-1.32) | ＜0.001* |  |
| **Incident coronary heart disease (n =33,426)** | | | | | | | |
| Men |  |  |  |  |  |  |  |
| Model 1^a^ | 1.00 (*ref*) | 0.93 (0.89-0.98) | 0.94 (0.90-0.99) | 1.05 (0.99-1.10) | 1.21 (1.13-1.29) | ＜0.001* | 0.03* |
| Model 2^b^ | 1.00 (*ref*) | 0.98 (0.93-1.02) | 1.02 (0.97-1.06) | 1.11 (1.05-1.17) | 1.21 (1.13-1.29) | ＜0.001* |  |
| Women |  |  |  |  |  |  |  |
| Model 1^a^ | 1.00 (*ref*) | 0.91 (0.88-0.95) | 0.90 (0.86-0.93) | 0.98 (0.94-1.03) | 1.14 (1.07-1.22) | 0.07 | 0.03* |
| Model 2^b^ | 1.00 (*ref*) | 0.96 (0.92-1.00) | 0.96 (0.92-1.00) | 1.04 (0.99-1.10) | 1.13 (1.06-1.21) | ＜0.001* |  |
| **Incident stroke (n = 9,706)** | | | | | | | |
| Men |  |  |  |  |  |  |  |
| Model 1^a^ | 1.00 (*ref*) | 0.96 (0.88-1.04) | 1.00 (0.92-1.09) | 1.03 (0.93-1.13) | 1.14 (1.00-1.29) | 0.06 | 0.90 |
| Model 2^b^ | 1.00 (*ref*) | 0.99 (0.91-1.08) | 1.06 (0.98-1.15) | 1.08 (0.97-1.19) | 1.14 (1.00-1.29) | 0.01* |  |
| Women |  |  |  |  |  |  |  |
| Model 1^a^ | 1.00 (*ref*) | 0.98 (0.91-1.06) | 0.97 (0.90-1.05) | 1.06 (0.97-1.16) | 1.17 (1.04-1.31) | 0.03* | 0.90 |
| Model 2^b^ | 1.00 (*ref*) | 1.02 (0.94-1.10) | 1.03 (0.96-1.11) | 1.13 (1.02-1.24) | 1.16 (1.03-1.31) | ＜0.01* |  |

**Table S3**. Hazard ratio (95% CIs) for cardiovascular diseases according to coffee intake among men and women in the UK Biobank

Model 1 was adjusted for ethnicity and baseline age. Model 2 was additionally adjusted for qualification, employment, income, smoking status, physical activity pattern, diet pattern, body mass index, systolic blood pressure, diastolic blood pressure, triglycerides, LDL-cholesterol, HDL-cholesterol, long-term illness status, alcohol consumption.

| Outcomes | Non-consumers | Tea intake (cups per day) | | | | p for trend | P  interaction |
| --- | --- | --- | --- | --- | --- | --- | --- |
|  |  | 0.5-1 | 2-3 | 4-5 | ≥6 |  |  |
|  | (*n*=67,722) | (*n*=53,742) | (*n*=136,180) | (*n*=117,562) | (*n*=86,416) |  |  |
| **Incident heart failure (n = 11,098)** | | | | | | | |
| Men |  |  |  |  |  |  |  |
| Model 1^a^ | 1.00 (*ref*) | 0.88 (0.79-0.98) | 0.93 (0.85-1.02) | 0.94 (0.86-1.03) | 1.16 (1.06-1.28) | ＜0.001* | 0.70 |
| Model 2^b^ | 1.00 (*ref*) | 0.94 (0.84-1.05) | 1.00 (0.91-1.09) | 0.97 (0.89-1.06) | 1.11 (1.02-1.22) | 0.02* |  |
| Women |  |  |  |  |  |  |  |
| Model 1^a^ | 1.00 (*ref*) | 0.85 (0.77-0.94) | 0.87 (0.80-0.94) | 0.97 (0.90-1.05) | 1.09 (1.00-1.19) | ＜0.001* | 0.68 |
| Model 2^b^ | 1.00 (*ref*) | 0.92 (0.83-1.02) | 0.92 (0.85-1.00) | 1.00 (0.92-1.08) | 1.05 (0.96-1.14) | 0.06 |  |
| **Incident coronary heart disease (n =33,426)** | | | | | | | |
| Men |  |  |  |  |  |  |  |
| Model 1^a^ | 1.00 (*ref*) | 0.90 (0.84-0.95) | 0.93 (0.89-0.98) | 0.97 (0.92-1.02) | 1.08 (1.02-1.14) | ＜0.001* | 0.04 * |
| Model 2^b^ | 1.00 (*ref*) | 0.94 (0.89-1.00) | 0.98 (0.93-1.03) | 0.99 (0.94-1.04) | 1.05 (0.99-1.10) | 0.02* |  |
| Women |  |  |  |  |  |  |  |
| Model 1^a^ | 1.00 (*ref*) | 0.91 (0.86-0.97) | 0.98 (0.94-1.03) | 1.03 (0.99-1.09) | 1.15 (1.10-1.21) | ＜0.001* | 0.05 * |
| Model 2^b^ | 1.00 (*ref*) | 0.96 (0.91-1.02) | 1.02 (0.98-1.07) | 1.05 (1.00-1.10) | 1.12 (1.06-1.18) | ＜0.001* |  |
| **Incident stroke (n = 9,706)** | | | | | | | |
| Men |  |  |  |  |  |  |  |
| Model 1^a^ | 1.00 (*ref*) | 0.99 (0.88-1.11) | 0.95 (0.86-1.04) | 0.92 (0.84-1.02) | 1.02 (0.92-1.12) | 0.79 | 0.28 |
| Model 2^b^ | 1.00 (*ref*) | 1.04 (0.92-1.16) | 0.98 (0.90-1.08) | 0.93 (0.85-1.03) | 0.98 (0.89-1.08) | 0.21 |  |
| Women |  |  |  |  |  |  |  |
| Model 1^a^ | 1.00 (*ref*) | 0.94 (0.84-1.04) | 0.90 (0.83-0.98) | 0.92 (0.84-1.01) | 1.07 (0.97-1.17) | 0.20 | 0.30 |
| Model 2^b^ | 1.00 (*ref*) | 0.99 (0.89-1.10) | 0.93 (0.85-1.01) | 0.92 (0.85-1.01) | 1.03 (0.94-1.13) | 0.95 |  |

**Table S4.** Hazard ratio (95% CIs) for cardiovascular diseases according to tea intake among men and women in the UK Biobank

Model 1 was adjusted for ethnicity and baseline age. Model 2 was additionally adjusted for qualification, employment, income, smoking status, physical activity pattern, diet pattern, body mass index, systolic blood pressure, diastolic blood pressure, triglycerides, LDL-cholesterol, HDL-cholesterol, long-term illness status, alcohol consumption.

**Table S5.** Hazard ratio (95% CIs) for cardiovascular diseases according to coffee and tea intake among men and women in the UK Biobank

| Outcomes | Non-consumers | Composite intake (cups per day) | | | | p for trend | p for sex interaction |
| --- | --- | --- | --- | --- | --- | --- | --- |
|  |  | 0.5-2 | ＞2-4 | ＞4-8 | ＞8 |  |  |
|  | (*n*=10,888) | (*n*=41,524) | (*n*=123,836) | (*n*=236,335) | (*n*=49,039) |  |  |
| **Incident heart failure (n = 11,098)** | | | | | | | |
| Men |  |  |  |  |  |  |  |
| Model 1^a^ | 1.00 (*ref*) | 1.11 (0.89-1.38) | 1.16 (0.95-1.42) | 1.14 (0.93-1.39) | 1.54 (1.25-1.90) | ＜0.001* | 0.39 |
| Model 2^b^ | 1.00 (*ref*) | 1.13 (0.91-1.41) | 1.24 (1.01-1.52) | 1.20 (0.98-1.46) | 1.48 (1.20-1.83) | ＜0.001* |  |
| Model 3^c^ | 1.00 (*ref*) | 1.13 (0.91-1.40) | 1.22 (0.99-1.49) | 1.17 (0.95-1.43) | 1.44 (1.16-1.78) | ＜0.001* |  |
| Women |  |  |  |  |  |  | 0.50 |
| Model 1^a^ | 1.00 (*ref*) | 1.09 (0.89-1.33) | 1.12 (0.93-1.35) | 1.19 (0.99-1.43) | 1.53 (1.26-1.85) | ＜0.001* |  |
| Model 2^b^ | 1.00 (*ref*) | 1.13 (0.92-1.38) | 1.21 (1.00-1.45) | 1.27 (1.05-1.52) | 1.46 (1.21-1.77) | ＜0.001* |  |
| Model 3^c^ | 1.00 (*ref*) | 1.13 (0.92-1.38) | 1.21 (1.00-1.45) | 1.26 (1.05-1.52) | 1.46 (1.20-1.77) | ＜0.001* | 0.50 |
| **Incident coronary heart disease (n =33,426)** | | | | | | | |
| Men |  |  |  |  |  |  |  |
| Model 1^a^ | 1.00 (*ref*) | 1.16 (1.02-1.32) | 1.27 (1.13-1.44) | 1.30 (1.15-1.46) | 1.60 (1.41-1.82) | ＜0.001* | 0.94 |
| Model 2^b^ | 1.00 (*ref*) | 1.19 (1.05-1.36) | 1.34 (1.19-1.52) | 1.35 (1.20-1.53) | 1.57 (1.38-1.78) | ＜0.001* |  |
| Model 3^c^ | 1.00 (*ref*) | 1.18 (1.03-1.34) | 1.30 (1.15-1.47) | 1.29 (1.14-1.46) | 1.48 (1.31-1.68) | ＜0.001* |  |
| Women |  |  |  |  |  |  | 0.93 |
| Model 1^a^ | 1.00 (*ref*) | 1.19 (1.05-1.34) | 1.26 (1.13-1.41) | 1.32 (1.18-1.47) | 1.60 (1.43-1.80) | ＜0.001* |  |
| Model 2^b^ | 1.00 (*ref*) | 1.21 (1.07-1.36) | 1.32 (1.18-1.48) | 1.36 (1.22-1.52) | 1.54 (1.38-1.73) | ＜0.001* |  |
| Model 3^c^ | 1.00 (*ref*) | 1.20 (1.07-1.35) | 1.29 (1.15-1.44) | 1.31 (1.17-1.46) | 1.48 (1.32-1.66) | ＜0.001* | 0.90 |
| **Incident stroke (n = 9,706)** | | | | | | | |
| Men |  |  |  |  |  |  |  |
| Model 1^a^ | 1.00 (*ref*) | 1.00 (0.80-1.25) | 1.07 (0.87-1.31) | 1.02 (0.83-1.24) | 1.22 (0.98-1.51) | 0.06 | 0.68 |
| Model 2^b^ | 1.00 (*ref*) | 1.00 (0.80-1.24) | 1.08 (0.88-1.33) | 1.02 (0.83-1.25) | 1.15 (0.93-1.43) | 0.24 |  |
| Model 3^c^ | 1.00 (*ref*) | 0.98 (0.79-1.23) | 1.04 (0.85-1.28) | 0.97 (0.79-1.19) | 1.09 (0.87-1.35) | 0.80 |  |
| Women |  |  |  |  |  |  | 0.76 |
| Model 1^a^ | 1.00 (*ref*) | 0.94 (0.78-1.13) | 0.83 (0.70-0.99) | 0.87 (0.73-1.03) | 1.14 (0.95-1.37) | ＜0.01* |  |
| Model 2^b^ | 1.00 (*ref*) | 0.93 (0.77-1.13) | 0.84 (0.71-1.01) | 0.87 (0.73-1.03) | 1.07 (0.90-1.29) | 0.08 |  |
| Model 3^c^ | 1.00 (*ref*) | 0.92 (0.76-1.11) | 0.81 (0.68-0.97) | 0.82 (0.69-0.98) | 1.01 (0.84-1.21) | 0.50 | 0.75 |

Model 1 was adjusted for ethnicity and baseline age. Model 2 was additionally adjusted for qualification, employment, income, smoking status, physical activity pattern, diet pattern, body mass index, systolic blood pressure, diastolic blood pressure, triglycerides, LDL-cholesterol, HDL-cholesterol, long-term illness status, alcohol consumption. Model 3 was additionally adjusted for water consumption.

**Table S6.** Baseline characteristics of the study population aged 60 and above

| **Characteristic** | **Women** | **Men** | **P values** |
| --- | --- | --- | --- |
| Number (n, %) | 92,927 (52.47) | 84,177 (47.53) | – |
| Age (years) | 64.57 (2.59) | 64.76 (2.60) | <0.001 |
| Ethnic (n, %) |  |  | <0.001 |
| White | 86,050 (92.60) | 190,487 (90.60) |  |
| Black or Black British | 6,478 (7.00) | 5,041 (6.00) |  |
| Other ethnic | 399 (0.40) | 576 (0.70) |  |
| Education, university or college (n, %) | 21,018 (22.6) | 23,511 (27.9) | <0.001 |
| Employment (n, %) |  |  | <0.001 |
| Working | 18,281 (19.70) | 24,737 (29.40) |  |
| Retired | 72,322 (77.8) | 55,111 (65.50) |  |
| Unemployment | 1,527 (1.60) | 3,497 (4.20) |  |
| None of the above | 797 (0.90) | 832 (1.00) |  |
| Income (n, %) |  |  | 0.843 |
| Less than £18,000 | 20,585 (22.20) | 18,463 (21.90) |  |
| £18,000 to £30,999 | 23,656 (25.50) | 21,486 (25.50) |  |
| £31,000 to £51,999 | 24,675 (26.60) | 22,354 (26.60) |  |
| £52,000 to £100,000 | 18,900 (20.30) | 17,220 (20.50) |  |
| £100,000 and above | 5,111 (5.50) | 4,654 (5.50) |  |
| Smoking status (n, %) |  |  | <0.001 |
| Never | 53,215 (57.30) | 34,726 (41.30) |  |
| Previous | 33,567 (36.10) | 41,199 (48.90) |  |
| Current | 6,145 (6.60) | 8,252 (9.80) |  |
| Healthy physical activity pattern (n, %) | 59,167 (63.70) | 53,339 (63.40) | 0.184 |
| Healthy diet pattern (n, %) | 19,152 (20.60) | 17,166 (20.40) | 0.261 |
| Body mass index (kg/m^2^) | 27.36 (4.90) | 27.86 (4.08) | <0.001 |
| Systolic blood pressure (mmHg) | 145.26 (20.07) | 147.65 (19.23) | <0.001 |
| Diastolic blood pressure (mmHg) | 81.19 (10.42) | 83.64 (10.52) | <0.001 |
| LDL-cholesterol (mmol/L) | 3.74 (0.90) | 3.34 (0.88) | <0.001 |
| HDL-cholesterol (mmol/L) | 1.61 (0.38) | 1.29 (0.32) | <0.001 |
| Townsend Deprivation Index | -1.39 (3.03) | -1.36 (3.06) | 0.020 |
| Alcohol consumption (n, %) |  |  | 0.041 |
| Never | 7,268 (7.80) | 6,326 (7.50) |  |
| Special occasions only | 20,986 (22.60) | 18,816 (22.40) |  |
| One to four times a week | 45,642 (49.10) | 41,642 (49.50) |  |
| Daily or almost daily | 19031 (20.50) | 17,393 (20.70) |  |
| Water consumption (n, %) | 2.73 (2.22) | 2.71 (2.26) | 0.259 |
| Coffee consumption (n, %) | 2.01 (2.07) | 2.01 (2.07) | 0.975 |
| Tea consumption (n, %) | 3.41 (2.86) | 3.41 (2.85) | 0.840 |
| Com-consumption (n, %) | 5.42 (3.04) | 5.43 (3.04) | 0.866 |

Values are shown as mean (standard deviation) for continuous variables and number (percentage) for categorical variables. Difference between women and men are compared using Student’s *t* test or chi-square test accordingly. Abbreviations: LDL-cholesterol, low-density lipoprotein cholesterol; HDL-cholesterol, high-density lipoprotein cholesterol.

**Table S7.** Hazard ratio (95% CIs) for cardiovascular diseases by water intake in individuals aged 60 and over in the UK Biobank

| Outcomes | Non-consumers | Water intake (cups per day) | | | | p for trend | p for sex interaction |
| --- | --- | --- | --- | --- | --- | --- | --- |
|  |  | 0.5-1 | 2-3 | 4-5 | ≥6 |  |  |
|  | (*n*=14,250) | (*n*=44,283) | (*n*=67,213) | (*n*=32,237) | (*n*=19,121) |  |  |
| **Incident heart failure (n = 4,403)** | | | | | | | |
| Men |  |  |  |  |  |  |  |
| Model 1^a^ | 1.00 (*ref*) | 0.93 (0.79-1.10) | 0.88 (0.75-1.03) | 0.85 (0.72-1.02) | 0.81 (0.67-0.99) | 0.02* | 0.47 |
| Model 2^b^ | 1.00 (*ref*) | 1.05 (0.89-1.24) | 1.02 (0.87-1.20) | 1.02 (0.86-1.22) | 0.99 (0.81-1.21) | 0.74 |  |
| Women |  |  |  |  |  |  |  |
| Model 1^a^ | 1.00 (*ref*) | 0.78 (0.67-0.91) | 0.74 (0.64-0.86) | 0.72 (0.62-0.85) | 0.71 (0.59-0.85) | ＜0.001* | 0.27 |
| Model 2^b^ | 1.00 (*ref*) | 0.87 (0.75-1.01) | 0.84 (0.72-0.97) | 0.83 (0.71-0.98) | 0.82 (0.68-0.99) | 0.05* |  |
| **Incident coronary heart disease (n =12,968)** | | | | | | | |
| Men |  |  |  |  |  |  |  |
| Model 1^a^ | 1.00 (*ref*) | 0.80 (0.73-0.88) | 0.76 (0.70-0.83) | 0.68 (0.62-0.75) | 0.70 (0.62-0.78) | ＜0.001* | 0.25 |
| Model 2^b^ | 1.00 (*ref*) | 0.87 (0.79-0.95) | 0.84 (0.77-0.92) | 0.77 (0.69-0.85) | 0.79 (0.71-0.89) | ＜0.001* |  |
| Women |  |  |  |  |  |  |  |
| Model 1^a^ | 1.00 (*ref*) | 0.81 (0.75-0.89) | 0.77 (0.71-0.84) | 0.77 (0.70-0.84) | 0.70 (0.63-0.78) | ＜0.001* | 0.49 |
| Model 2^b^ | 1.00 (*ref*) | 0.87 (0.80-0.95) | 0.83 (0.77-0.91) | 0.85 (0.77-0.93) | 0.78 (0.70-0.87) | ＜0.001* |  |
| **Incident stroke (n = 3,793)** | | | | | | | |
| Men |  |  |  |  |  |  |  |
| Model 1^a^ | 1.00 (*ref*) | 0.88 (0.74-1.05) | 0.86 (0.73-1.01) | 0.71 (0.59-0.86) | 0.71 (0.58-0.88) | ＜0.001* | 0.90 |
| Model 2^b^ | 1.00 (*ref*) | 0.96 (0.81-1.15) | 0.96 (0.81-1.13) | 0.81 (0.67-0.98) | 0.83 (0.67-1.03) | 0.01* |  |
| Women |  |  |  |  |  |  |  |
| Model 1^a^ | 1.00 (*ref*) | 0.79 (0.67-0.93) | 0.73 (0.63-0.86) | 0.71 (0.60-0.85) | 0.66 (0.54-0.81) | ＜0.001* | 0.91 |
| Model 2^b^ | 1.00 (*ref*) | 0.84 (0.71-0.99) | 0.78 (0.67-0.91) | 0.76 (0.64-0.91) | 0.71 (0.58-0.87) | ＜0.001* |  |

Model 1 was adjusted for ethnic and baseline age. Model 2 was additionally adjusted for qualification, employment, income, smoking status, physical activity pattern, diet pattern, BMI, SBP, DBP, triglycerides, LDL, HDL, longtime illness status, alcohol consumption status, coffee consumption status, tea consumption status.

**Table S8.** Hazard ratio (95% CIs) for cardiovascular diseases by coffee intake in individuals aged 60 and over in the UK Biobank

| Outcomes | Non-consumers | Coffee intake (cups per day) | | | | p for trend | p for sex interaction |
| --- | --- | --- | --- | --- | --- | --- | --- |
|  |  | 0.5-1 | 2-3 | 4-5 | ≥6 |  |  |
|  | (*n*=38,897) | (*n*=48,544) | (*n*=55,376) | (*n*=23,669) | (*n*=10,618) |  |  |
| **Incident heart failure (n = 4,403)** | | | | | | | |
| Men |  |  |  |  |  |  |  |
| Model 1^a^ | 1.00 (*ref*) | 0.87 (0.77-0.98) | 0.93 (0.83-1.05) | 0.96 (0.83-1.11) | 0.93 (0.76-1.13) | 0.67 | 0.49 |
| Model 2^b^ | 1.00 (*ref*) | 0.93 (0.83-1.06) | 1.03 (0.92-1.16) | 1.04 (0.90-1.21) | 0.94 (0.77-1.15) | 0.61 |  |
| Women |  |  |  |  |  |  |  |
| Model 1^a^ | 1.00 (*ref*) | 0.87 (0.78-0.98) | 0.86 (0.77-0.96) | 0.99 (0.86-1.13) | 1.11 (0.93-1.32) | 0.59 | 0.53 |
| Model 2^b^ | 1.00 (*ref*) | 0.93 (0.83-1.05) | 0.96 (0.86-1.08) | 1.08 (0.94-1.24) | 1.10 (0.93-1.31) | 0.09 |  |
| **Incident coronary heart disease (n =12,968)** | | | | | | | |
| Men |  |  |  |  |  |  |  |
| Model 1^a^ | 1.00 (*ref*) | 0.93 (0.87-1.00) | 0.96 (0.89-1.03) | 1.10 (1.01-1.19) | 1.21 (1.08-1.34) | ＜0.001* | 0.06 |
| Model 2^b^ | 1.00 (*ref*) | 0.98 (0.92-1.06) | 1.04 (0.96-1.11) | 1.17 (1.07-1.27) | 1.22 (1.10-1.36) | ＜0.001* |  |
| Women |  |  |  |  |  |  |  |
| Model 1^a^ | 1.00 (*ref*) | 0.88 (0.82-0.94) | 0.88 (0.83-0.94) | 0.98 (0.91-1.06) | 1.11 (1.00-1.23) | 0.29 | 0.05 * |
| Model 2^b^ | 1.00 (*ref*) | 0.92 (0.86-0.99) | 0.95 (0.89-1.02) | 1.04 (0.96-1.13) | 1.11 (1.00-1.22) | 0.03* |  |
| **Incident stroke (n = 3,793)** | | | | | | | |
| Men |  |  |  |  |  |  |  |
| Model 1^a^ | 1.00 (*ref*) | 0.99 (0.87-1.13) | 1.01 (0.89-1.15) | 1.16 (0.99-1.35) | 1.08 (0.88-1.33) | 0.10 | 0.82 |
| Model 2^b^ | 1.00 (*ref*) | 1.03 (0.90-1.18) | 1.08 (0.95-1.23) | 1.22 (1.04-1.43) | 1.09 (0.88-1.34) | 0.03* |  |
| Women |  |  |  |  |  |  |  |
| Model 1^a^ | 1.00 (*ref*) | 0.91 (0.81-1.04) | 0.90 (0.79-1.02) | 1.10 (0.95-1.27) | 1.23 (1.02-1.48) | 0.05* | 0.85 |
| Model 2^b^ | 1.00 (*ref*) | 0.94 (0.83-1.07) | 0.94 (0.83-1.07) | 1.15 (0.99-1.34) | 1.22 (1.01-1.47) | 0.01* |  |

Model 1 was adjusted for ethnic and baseline age. Model 2 was additionally adjusted for qualification, employment, income, smoking status, physical activity pattern, diet pattern, BMI, SBP, DBP, triglycerides, LDL, HDL, longtime illness status, alcohol consumption status.

**Table S9.** Hazard ratio (95% CIs) for cardiovascular diseases by tea intake in individuals aged 60 and over in the UK Biobank

| Outcomes | Non-consumers | Tea intake (cups per day) | | | | p for trend | p for sex interaction |
| --- | --- | --- | --- | --- | --- | --- | --- |
|  |  | 0.5-1 | 2-3 | 4-5 | ≥6 |  |  |
|  | (*n*=25,843) | (*n*=20,440) | (*n*=52,373) | (*n*=45,290) | (*n*=33,158) |  |  |
| **Incident heart failure (n = 4,403)** | | | | | | | |
| Men |  |  |  |  |  |  |  |
| Model 1^a^ | 1.00 (*ref*) | 0.95 (0.80-1.14) | 1.01 (0.88-1.17) | 1.01 (0.87-1.16) | 1.22 (1.06-1.42) | ＜0.01* | 0.92 |
| Model 2^b^ | 1.00 (*ref*) | 1.03 (0.87-1.23) | 1.09 (0.95-1.25) | 1.04 (0.90-1.20) | 1.17 (1.01-1.36) | 0.06 |  |
| Women |  |  |  |  |  |  |  |
| Model 1^a^ | 1.00 (*ref*) | 0.85 (0.72-1.00) | 0.91 (0.80-1.04) | 0.98 (0.86-1.12) | 1.15 (1.00-1.32) | ＜0.01* | 0.97 |
| Model 2^b^ | 1.00 (*ref*) | 0.92 (0.78-1.09) | 0.98 (0.86-1.11) | 1.01 (0.89-1.16) | 1.11 (0.97-1.28) | 0.05* |  |
| **Incident coronary heart disease (n =12,968)** | | | | | | | |
| Men |  |  |  |  |  |  |  |
| Model 1^a^ | 1.00 (*ref*) | 0.89 (0.81-0.99) | 0.92 (0.85-0.99) | 0.96 (0.89-1.04) | 1.05 (0.96-1.14) | 0.07 | 0.03 * |
| Model 2^b^ | 1.00 (*ref*) | 0.95 (0.86-1.05) | 0.96 (0.89-1.04) | 0.98 (0.91-1.07) | 1.02 (0.93-1.11) | 0.47 |  |
| Women |  |  |  |  |  |  |  |
| Model 1^a^ | 1.00 (*ref*) | 0.99 (0.90-1.09) | 1.04 (0.96-1.13) | 1.10 (1.02-1.19) | 1.19 (1.10-1.30) | ＜0.001* | 0.02 * |
| Model 2^b^ | 1.00 (*ref*) | 1.05 (0.95-1.16) | 1.09 (1.01-1.18) | 1.13 (1.04-1.22) | 1.17 (1.07-1.27) | ＜0.001* |  |
| **Incident stroke (n = 3,793)** | | | | | | | |
| Men |  |  |  |  |  |  |  |
| Model 1^a^ | 1.00 (*ref*) | 1.01 (0.84-1.21) | 0.98 (0.84-1.13) | 0.99 (0.85-1.15) | 1.13 (0.96-1.32) | 0.17 | 0.24 |
| Model 2^b^ | 1.00 (*ref*) | 1.06 (0.89-1.28) | 1.02 (0.88-1.18) | 1.01 (0.86-1.17) | 1.09 (0.93-1.27) | 0.49 |  |
| Women |  |  |  |  |  |  |  |
| Model 1^a^ | 1.00 (*ref*) | 0.92 (0.78-1.09) | 0.82 (0.71-0.94) | 0.89 (0.77-1.02) | 0.98 (0.84-1.13) | 0.79 | 0.28 |
| Model 2^b^ | 1.00 (*ref*) | 0.97 (0.82-1.15) | 0.84 (0.73-0.97) | 0.89 (0.77-1.03) | 0.95 (0.82-1.10) | 0.37 |  |

Model 1 was adjusted for ethnic and baseline age. Model 2 was additionally adjusted for qualification, employment, income, smoking status, physical activity pattern, diet pattern, BMI, SBP, DBP, triglycerides, LDL, HDL, longtime illness status, alcohol consumption status.

**Table S10.** Hazard ratio (95% CIs) for cardiovascular diseases by coffee and tea intake in individuals aged 60 and over in the UK Biobank

| Outcomes | Non-consumers | Composite intake (cups per day) | | | | p for trend | p for sex interaction |
| --- | --- | --- | --- | --- | --- | --- | --- |
|  |  | 0.5-2 | ＞2-4 | ＞4-8 | ＞8 |  |  |
|  | (n=4,067) | (n=15,858) | (n=47,724) | (n=90,790) | (n=18,665) |  |  |
| **Incident heart failure (n = 4,403)** | | | | | | | |
| Men |  |  |  |  |  |  |  |
| Model 1^a^ | 1.00 (*ref*) | 1.23 (0.86-1.77) | 1.32 (0.94-1.85) | 1.29 (0.93-1.80) | 1.64 (1.16-2.32) | ＜0.01* | 0.66 |
| Model 2^b^ | 1.00 (*ref*) | 1.23 (0.86-1.77) | 1.37 (0.98-1.93) | 1.33 (0.95-1.87) | 1.55 (1.09-2.20) | 0.02* |  |
| Women |  |  |  |  |  |  |  |
| Model 1^a^ | 1.00 (*ref*) | 1.01 (0.73-1.39) | 1.12 (0.83-1.51) | 1.12 (0.84-1.50) | 1.48 (1.09-2.02) | ＜0.001* | 0.70 |
| Model 2^b^ | 1.00 (*ref*) | 1.03 (0.75-1.42) | 1.18 (0.88-1.59) | 1.15 (0.86-1.55) | 1.37 (1.00-1.87) | 0.01* |  |
| **Incident coronary heart disease (n =12,968)** | | | | | | | |
| Men |  |  |  |  |  |  |  |
| Model 1^a^ | 1.00 (*ref*) | 1.14 (0.93-1.40) | 1.21 (1.00-1.46) | 1.24 (1.03-1.50) | 1.57 (1.29-1.91) | ＜0.001* | 0.52 |
| Model 2^b^ | 1.00 (*ref*) | 1.15 (0.94-1.41) | 1.23 (1.01-1.49) | 1.23 (1.01-1.48) | 1.45 (1.18-1.76) | ＜0.001* |  |
| Women |  |  |  |  |  |  |  |
| Model 1^a^ | 1.00 (*ref*) | 1.21 (0.99-1.48) | 1.30 (1.08-1.57) | 1.38 (1.14-1.65) | 1.67 (1.38-2.03) | ＜0.001* | 0.59 |
| Model 2^b^ | 1.00 (*ref*) | 1.24 (1.02-1.52) | 1.34 (1.11-1.62) | 1.39 (1.16-1.68) | 1.57 (1.29-1.90) | ＜0.001* |  |
| **Incident stroke (n = 3,793)** | | | | | | | |
| Men |  |  |  |  |  |  |  |
| Model 1^a^ | 1.00 (*ref*) | 1.22 (0.82-1.82) | 1.45 (0.99-2.10) | 1.41 (0.98-2.04) | 1.75 (1.19-2.58) | 0.001* | 0.17 |
| Model 2^b^ | 1.00 (*ref*) | 1.19 (0.80-1.78) | 1.41 (0.97-2.06) | 1.35 (0.93-1.96) | 1.55 (1.05-2.29) | 0.03* |  |
| Women |  |  |  |  |  |  |  |
| Model 1^a^ | 1.00 (*ref*) | 0.87 (0.65-1.17) | 0.71 (0.54-0.93) | 0.80 (0.61-1.05) | 1.00 (0.75-1.33) | 0.14 | 0.17 |
| Model 2^b^ | 1.00 (*ref*) | 0.85 (0.63-1.15) | 0.68 (0.51-0.89) | 0.75 (0.57-0.98) | 0.87 (0.65-1.17) | 0.88 |  |

Model 1 was adjusted for ethnic and baseline age. Model 2 was additionally adjusted for qualification, employment, income, smoking status, physical activity pattern, diet pattern, BMI, SBP, DBP, triglycerides, LDL, HDL, longtime illness status, alcohol consumption status, water consumption status.

**Table S11.** Baseline characteristics of the study population without long time illness

| **Characteristic** | **Women** | **Men** | **P-values** |
| --- | --- | --- | --- |
| Number (n, %) | 176,617 (54.50) | 147,469 (45.50) | – |
| Age (years) | 56.31 (8.00) | 56.72 (8.20) | <0.001 |
| Ethnic (n, %) |  |  | <0.001 |
| White | 159,260 (90.20) | 133,560 (90.60) |  |
| Black or Black British | 16,563 (9.40) | 12,921 (8.80) |  |
| Other ethnic | 794 (0.40) | 988 (0.70) |  |
| Education, university or college (n, %) | 37,314 (31.00) | 49,484 (33.56) | <0.001 |
| Employment (n, %) |  |  | <0.001 |
| Working | 98,475 (55.80) | 89,889 (61.00) |  |
| Retired | 61,391 (34.80) | 45,745 (31.00) |  |
| Unemployment | 14,762 (8.40) | 10,155 (6.90) |  |
| None of the above | 1,989 (1.10) | 1,680 (1.10) |  |
| Income (n, %) |  |  | 0.872 |
| Less than £18,000 | 31,769 (18.00) | 26,349 (17.90) |  |
| £18,000 to £30,999 | 43,983 (24.90) | 36,673 (24.90) |  |
| £31,000 to £51,999 | 49,189 (27.90) | 41,104 (27.90) |  |
| £52,000 to £100,000 | 40,412 (22.90) | 33,867 (23.00) |  |
| £100,000 and above | 11,264 (6.40) | 9,476 (6.40) |  |
| Smoking status (n, %) |  |  | <0.001 |
| Never | 105,478 (59.70) | 72,542 (49.20) |  |
| Previous | 55,296 (31.30) | 56,415 (38.30) |  |
| Current | 15,843 (9.00) | 18,512 (12.60) |  |
| Body mass index (kg/m^2^) | 27.07 (5.18) | 27.83 (4.24) | <0.001 |
| Systolic blood pressure (mmHg) | 137.21 (20.23) | 142.70 (18.58) | <0.001 |
| Diastolic blood pressure (mmHg) | 80.70 (10.58) | 84.03 (10.56) | <0.001 |
| LDL-cholesterol (mmol/L) | 3.63 (0.87) | 3.48 (0.86) | <0.001 |
| HDL-cholesterol (mmol/L) | 1.59 (0.37) | 1.28 (0.32) | <0.001 |
| Townsend Deprivation Index | -1.57 (2.93) | -1.53 (2.95) | 0.002 |
| Healthy diet pattern (n, %) | 36,596 (20.70) | 30,477 (20.70) |  |
| Alcohol consumption (n, %) |  |  | 0.022 |
| Never | 10,941 (6.20) | 9,005 (6.10) |  |
| Special occasions only | 37,035 (21.00) | 30,364 (20.60) |  |
| One to four times a week | 91,017 (51.50) | 76,645 (52.00) |  |
| Daily or almost daily | 37,624 (21.30) | 31,455 (21.30) |  |
| Water consumption (n, %) | 2.72 (2.22) | 2.72 (2.22) | 0.604 |
| Coffee consumption (n, %) | 2.00 (2.00) | 2.02 (2.02) | 0.002 |
| Tea consumption (n, %) | 3.35 (2.74) | 3.36 (2.76) | 0.405 |
| Com-consumption (n, %) | 5.35 (2.88) | 5.38 (2.92) | 0.003 |

Values are shown as mean (standard deviation) for continuous variables and number (percentage) for categorical variables. Difference between women and men are compared using Student’s t test or chi-square test accordingly. Abbreviations: LDL-cholesterol, low-density lipoprotein cholesterol; HDL-cholesterol, high-density lipoprotein cholesterol.

**Table S12.** Hazard ratio (95% CIs) for cardiovascular diseases by water intake in individuals without long time illness in the UK Biobank

| Outcomes | Non-consumers | Water intake (cups per day) | | | | p for trend | p for sex interaction |
| --- | --- | --- | --- | --- | --- | --- | --- |
|  |  | 0.5-1 | 2-3 | 4-5 | ≥6 |  |  |
|  | (*n*=25,092) | (*n*=81,472) | (*n*=123,581) | (*n*=59,235) | (*n*=34,706) |  |  |
| **Incident heart failure (n = 5,325)** | | | | | | | |
| Men |  |  |  |  |  |  |  |
| Model 1a | 1.00 (ref) | 0.80 (0.69-0.92) | 0.80 (0.69-0.92) | 0.65 (0.55-0.76) | 0.65 (0.54-0.78) | ＜0.001* | 0.19 |
| Model 2b | 1.00 (ref) | 0.86 (0.74-1.00) | 0.90 (0.78-1.04) | 0.78 (0.66-0.91) | 0.81 (0.67-0.98) | 0.02* |  |
| Women |  |  |  |  |  |  |  |
| Model 1a | 1.00 (ref) | 0.86 (0.75-0.99) | 0.86 (0.75-0.98) | 0.78 (0.67-0.91) | 0.72 (0.61-0.85) | ＜0.001* | 0.22 |
| Model 2b | 1.00 (ref) | 0.91 (0.79-1.05) | 0.95 (0.83-1.09) | 0.90 (0.78-1.05) | 0.88 (0.74-1.04) | 0.24 |  |
| **Incident coronary heart disease (n =18,532)** | | | | | | | |
| Men |  |  |  |  |  |  |  |
| Model 1a | 1.00 (ref) | 0.85 (0.78-0.92) | 0.79 (0.73-0.85) | 0.67 (0.61-0.73) | 0.67 (0.60-0.73) | ＜0.001* | 0.50 |
| Model 2b | 1.00 (ref) | 0.89 (0.82-0.96) | 0.85 (0.79-0.92) | 0.74 (0.68-0.81) | 0.76 (0.69-0.84) | ＜0.001* |  |
| Women |  |  |  |  |  |  |  |
| Model 1a | 1.00 (ref) | 0.86 (0.80-0.93) | 0.80 (0.74-0.86) | 0.73 (0.67-0.79) | 0.66 (0.60-0.72) | ＜0.001* | 0.54 |
| Model 2b | 1.00 (ref) | 0.90 (0.83-0.97) | 0.86 (0.80-0.92) | 0.80 (0.74-0.87) | 0.75 (0.68-0.82) | ＜0.001* |  |
| **Incident stroke (n = 5,600)** | | | | | | | |
| Men |  |  |  |  |  |  |  |
| Model 1a | 1.00 (ref) | 0.78 (0.68-0.90) | 0.71 (0.62-0.81) | 0.66 (0.57-0.77) | 0.62 (0.52-0.74) | ＜0.001* | 0.21 |
| Model 2b | 1.00 (ref) | 0.83 (0.72-0.95) | 0.77 (0.67-0.88) | 0.74 (0.63-0.86) | 0.70 (0.59-0.84) | 0.27 |  |
| Women |  |  |  |  |  |  |  |
| Model 1a | 1.00 (ref) | 0.89 (0.77-1.01) | 0.80 (0.70-0.91) | 0.73 (0.63-0.85) | 0.76 (0.65-0.90) | ＜0.001* | 0.24 |
| Model 2b | 1.00 (ref) | 0.93 (0.82-1.07) | 0.86 (0.75-0.98) | 0.80 (0.69-0.92) | 0.84 (0.71-1.00) | ＜0.01* |  |

Model 1 was adjusted for ethnic and baseline age. Model 2 was additionally adjusted for qualification, employment, income, smoking status, physical activity pattern, diet pattern, BMI, SBP, DBP, triglycerides, LDL, HDL, longtime illness status, alcohol consumption status, coffee consumption status, tea consumption status.

**Table S13.** Hazard ratio (95% CIs) for cardiovascular diseases by coffee intake in individuals without long time illness in the UK Biobank

| Outcomes | Non-consumers | Coffee intake (cups per day) | | | | p for trend | p for sex interaction |
| --- | --- | --- | --- | --- | --- | --- | --- |
|  |  | 0.5-1 | 2-3 | 4-5 | ≥6 |  |  |
|  | (*n*=68,508) | (*n*=89,337) | (*n*=104,025) | (*n*=43,690) | (*n*=18,526) |  |  |
| **Incident heart failure (n = 5,325)** | | | | | | | |
| Men |  |  |  |  |  |  |  |
| Model 1^a^ | 1.00 (*ref*) | 1.01 (0.90-1.14) | 1.08 (0.96-1.21) | 1.08 (0.94-1.25) | 1.34 (1.13-1.59) | ＜0.01* | 0.92 |
| Model 2^b^ | 1.00 (*ref*) | 1.03 (0.92-1.16) | 1.12 (1.00-1.26) | 1.13 (0.99-1.31) | 1.35 (1.13-1.60) | ＜0.001* |  |
| Women |  |  |  |  |  |  |  |
| Model 1^a^ | 1.00 (*ref*) | 0.98 (0.88-1.09) | 1.03 (0.93-1.14) | 1.12 (0.99-1.27) | 1.27 (1.09-1.50) | ＜0.01* | 0.93 |
| Model 2^b^ | 1.00 (*ref*) | 0.99 (0.89-1.10) | 1.05 (0.95-1.17) | 1.16 (1.02-1.31) | 1.29 (1.09-1.51) | ＜0.001* |  |
| **Incident coronary heart disease (n =18,532)** | | | | | | | |
| Men |  |  |  |  |  |  |  |
| Model 1^a^ | 1.00 (*ref*) | 0.94 (0.88-1.00) | 0.96 (0.90-1.02) | 1.04 (0.97-1.12) | 1.23 (1.12-1.35) | ＜0.001* | 0.18 |
| Model 2^b^ | 1.00 (*ref*) | 0.95 (0.90-1.02) | 0.98 (0.93-1.04) | 1.06 (0.99-1.14) | 1.23 (1.12-1.34) | ＜0.001* |  |
| Women |  |  |  |  |  |  |  |
| Model 1^a^ | 1.00 (*ref*) | 0.94 (0.89-1.00) | 0.94 (0.89-1.00) | 0.99 (0.93-1.06) | 1.11 (1.02-1.22) | 0.19 | 0.10 |
| Model 2^b^ | 1.00 (*ref*) | 0.95 (0.90-1.01) | 0.96 (0.91-1.01) | 1.02 (0.95-1.09) | 1.11 (1.02-1.21) | 0.07 |  |
| **Incident stroke (n = 5,600)** | | | | | | | |
| Men |  |  |  |  |  |  |  |
| Model 1^a^ | 1.00 (*ref*) | 1.00 (0.90-1.12) | 1.03 (0.93-1.15) | 1.05 (0.92-1.20) | 1.14 (0.95-1.35) | 0.15 | 0.52 |
| Model 2^b^ | 1.00 (*ref*) | 1.01 (0.90-1.13) | 1.06 (0.94-1.18) | 1.08 (0.94-1.23) | 1.13 (0.95-1.35) | 0.08 |  |
| Women |  |  |  |  |  |  |  |
| Model 1^a^ | 1.00 (*ref*) | 0.99 (0.89-1.09) | 0.95 (0.86-1.04) | 1.00 (0.88-1.13) | 1.14 (0.97-1.34) | 0.54 | 0.52 |
| Model 2^b^ | 1.00 (*ref*) | 1.00 (0.91-1.11) | 0.97 (0.88-1.08) | 1.04 (0.92-1.18) | 1.15 (0.98-1.35) | 0.23 |  |

Model 1 was adjusted for ethnic and baseline age. Model 2 was additionally adjusted for qualification, employment, income, smoking status, physical activity pattern, diet pattern, BMI, SBP, DBP, triglycerides, LDL, HDL, longtime illness status, alcohol consumption status, coffee consumption status, tea consumption status.

**Table S14.** Hazard ratio (95% CIs) for cardiovascular diseases by tea intake in individuals without long time illness in the UK Biobank

| Outcomes | Non-consumers | Tea intake (cups per day) | | | | p for trend | p for sex interaction |
| --- | --- | --- | --- | --- | --- | --- | --- |
|  |  | 0.5-1 | 2-3 | 4-5 | ≥6 |  |  |
|  | (*n*=46,148) | (*n*=38,543) | (*n*=98,433) | (*n*=83,181) | (*n*=57,781) |  |  |
| **Incident heart failure (n = 5,325)** | | | | | | | |
| Men |  |  |  |  |  |  |  |
| Model 1^a^ | 1.00 (*ref*) | 0.94 (0.80-1.10) | 0.94 (0.83-1.07) | 0.97 (0.85-1.10) | 1.20 (1.04-1.37) | ＜0.01* | 0.84 |
| Model 2^b^ | 1.00 (*ref*) | 0.98 (0.83-1.15) | 0.96 (0.84-1.09) | 0.96 (0.84-1.10) | 1.16 (1.01-1.33) | ＜0.001* |  |
| Women |  |  |  |  |  |  |  |
| Model 1^a^ | 1.00 (*ref*) | 0.88 (0.76-1.02) | 0.95 (0.84-1.07) | 1.06 (0.94-1.20) | 1.13 (1.00-1.28) | ＜0.01* | 0.92 |
| Model 2^b^ | 1.00 (*ref*) | 0.91 (0.78-1.05) | 0.95 (0.85-1.07) | 1.05 (0.93-1.18) | 1.09 (0.96-1.24) | 0.02* |  |
| **Incident coronary heart disease (n =18,532)** | | | | | | | |
| Men |  |  |  |  |  |  |  |
| Model 1^a^ | 1.00 (*ref*) | 0.92 (0.85-1.00) | 0.97 (0.90-1.04) | 0.98 (0.91-1.05) | 1.10 (1.02-1.18) | ＜0.01* | ＜0.05 * |
| Model 2^b^ | 1.00 (*ref*) | 0.95 (0.87-1.03) | 0.98 (0.91-1.05) | 0.97 (0.91-1.04) | 1.08 (1.00-1.16) | 0.04* |  |
| Women |  |  |  |  |  |  |  |
| Model 1^a^ | 1.00 (*ref*) | 1.00 (0.92-1.08) | 1.05 (0.99-1.13) | 1.12 (1.05-1.20) | 1.22 (1.14-1.31) | ＜0.001* | ＜0.05 * |
| Model 2^b^ | 1.00 (*ref*) | 1.02 (0.94-1.11) | 1.06 (0.99-1.13) | 1.11 (1.04-1.19) | 1.19 (1.11-1.28) | ＜0.001* |  |
| **Incident stroke (n = 5,600)** | | | | | | | |
| Men |  |  |  |  |  |  |  |
| Model 1^a^ | 1.00 (*ref*) | 0.99 (0.85-1.15) | 0.98 (0.86-1.11) | 0.95 (0.83-1.08) | 1.06 (0.93-1.22) | 0.60 | 0.41 |
| Model 2^b^ | 1.00 (*ref*) | 1.02 (0.87-1.18) | 0.98 (0.87-1.11) | 0.93 (0.82-1.06) | 1.03 (0.90-1.18) | 0.89 |  |
| Women |  |  |  |  |  |  |  |
| Model 1^a^ | 1.00 (*ref*) | 0.96 (0.83-1.11) | 0.98 (0.87-1.10) | 0.98 (0.87-1.10) | 1.12 (0.99-1.27) | 0.07 | 0.44 |
| Model 2^b^ | 1.00 (*ref*) | 0.99 (0.86-1.14) | 0.98 (0.88-1.10) | 0.96 (0.85-1.08) | 1.08 (0.95-1.22) | 0.37 |  |

Model 1 was adjusted for ethnic and baseline age. Model 2 was additionally adjusted for qualification, employment, income, smoking status, physical activity pattern, diet pattern, BMI, SBP, DBP, triglycerides, LDL, HDL, longtime illness status, alcohol consumption status, coffee consumption status, tea consumption status.

**Table S15.** Hazard ratio (95% CIs) for cardiovascular diseases by coffee and tea intake in individuals without long time illness in the UK Biobank

| Outcomes | Non-consumers | Composite intake (cups per day) | | | | p for trend | p for sex interaction |
| --- | --- | --- | --- | --- | --- | --- | --- |
|  |  | 0.5-2 | ＞2-4 | ＞4-8 | ＞8 |  |  |
|  | (*n*=7,131) | (*n*=28,809) | (*n*=88,869) | (*n*=167,170) | (*n*=32,107) |  |  |
| **Incident heart failure (n = 5,325)** | | | | | | | |
| Men |  |  |  |  |  |  |  |
| Model 1^a^ | 1.00 (*ref*) | 1.27 (0.89-1.81) | 1.37 (0.98-1.90) | 1.42 (1.02-1.97) | 1.82 (1.30-2.56) | ＜0.001* | 0.43 |
| Model 2^b^ | 1.00 (*ref*) | 1.20 (0.85-1.71) | 1.27 (0.91-1.78) | 1.30 (0.93-1.81) | 1.60 (1.14-2.26) | 0.01* |  |
| Women |  |  |  |  |  |  |  |
| Model 1^a^ | 1.00 (*ref*) | 1.01 (0.75-1.36) | 1.10 (0.84-1.45) | 1.22 (0.93-1.61) | 1.60 (1.21-2.13) | ＜0.001* | 0.49 |
| Model 2^b^ | 1.00 (*ref*) | 0.96 (0.71-1.29) | 1.04 (0.79-1.38) | 1.14 (0.87-1.50) | 1.45 (1.09-1.93) | ＜0.001* |  |
| **Incident coronary heart disease (n =18,532)** | | | | | | | |
| Men |  |  |  |  |  |  |  |
| Model 1^a^ | 1.00 (*ref*) | 1.16 (0.97-1.39) | 1.27 (1.07-1.50) | 1.31 (1.11-1.55) | 1.56 (1.31-1.86) | ＜0.001* | 0.71 |
| Model 2^b^ | 1.00 (*ref*) | 1.13 (0.94-1.35) | 1.19 (1.01-1.42) | 1.20 (1.01-1.42) | 1.38 (1.16-1.65) | ＜0.001* |  |
| Women |  |  |  |  |  |  |  |
| Model 1^a^ | 1.00 (*ref*) | 1.18 (1.00-1.38) | 1.24 (1.06-1.44) | 1.31 (1.13-1.53) | 1.59 (1.35-1.86) | ＜0.001* | 0.80 |
| Model 2^b^ | 1.00 (ref) | 1.13 (0.96-1.33) | 1.15 (0.99-1.35) | 1.19 (1.02-1.39) | 1.40 (1.19-1.64) | ＜0.001* |  |
| **Incident stroke (n = 5,600)** | | | | | | | |
| Men |  |  |  |  |  |  |  |
| Model 1^a^ | 1.00 (*ref*) | 1.05 (0.76-1.44) | 1.22 (0.91-1.64) | 1.15 (0.86-1.54) | 1.34 (0.98-1.82) | 0.06 | 0.62 |
| Model 2^b^ | 1.00 (*ref*) | 0.99 (0.72-1.36) | 1.11 (0.82-1.49) | 1.01 (0.75-1.36) | 1.14 (0.83-1.56) | 0.71 |  |
| Women |  |  |  |  |  |  |  |
| Model 1^a^ | 1.00 (*ref*) | 0.85 (0.67-1.08) | 0.75 (0.60-0.94) | 0.79 (0.64-0.99) | 1.02 (0.80-1.29) | 0.18 | 0.57 |
| Model 2^b^ | 1.00 (*ref*) | 0.81 (0.64-1.04) | 0.70 (0.56-0.88) | 0.72 (0.57-0.89) | 0.88 (0.70-1.13) | 0.57 |  |

Model 1 was adjusted for ethnic and baseline age. Model 2 was additionally adjusted for qualification, employment, income, smoking status, physical activity pattern, diet pattern, BMI, SBP, DBP, triglycerides, LDL, HDL, longtime illness status, alcohol consumption status, water consumption status.
